# Identification of the hsa_circ_0001314-Related ceRNA Network in Breast Cancer With Bioinformatics Analysis

**DOI:** 10.1155/ijog/4668425

**Published:** 2024-11-25

**Authors:** Yuge Ma, Bing Zhang, Liping Wang, Wei Rong, Ting Liu

**Affiliations:** Department of Pathology, Qiqihar Medical University, Qiqihar, China

**Keywords:** breast cancer, ceRNA, circRNA, GEO

## Abstract

Circular RNA (circRNA) serves as a competitive endogenous RNA (ceRNA) that plays a pivotal role in the initiation and progression of breast cancer (BC). However, compared to other noncoding RNAs (ncRNAs), research on circRNA in BC is still in its infancy. Through the analysis of circRNA datasets in the GEO database, hsa_circ_0001314, which is upregulated in BC, was selected as the focus of this study. RT-qPCR analysis showed that hsa_circ_0001314 was significantly upregulated in BC tissues and cells. Subsequently, the biological functions of hsa_circ_0001314 in BC cells were examined through CCK-8, wound healing, transwell invasion, and flow cytometry analyses. The research demonstrated that knocking down the expression level of hsa_circ_0001314 significantly inhibited cell proliferation, migration, and invasion abilities while notably promoting cell apoptosis. Bioinformatics methods were used to predict downstream miRNAs and mRNAs that may interact with hsa_circ_0001314, constructing a ceRNA regulatory network related to hsa_circ_0001314. RT-qPCR confirmed that hsa_circ_0001314 functions as a sponge for hsa-miR-548aj-3p, competitively binding to hsa-miR-548aj-3p to activate the MAPK signaling pathway and regulate the expression of MAPK8 and MAP3K1. The findings uncover the potential of hsa_circ_0001314 as a novel prognostic biomarker and therapeutic target for BC patients.

## 1. Introduction

Breast cancer (BC) is the most frequent disease among women and the primary cause of cancer-related deaths [1]. Despite improvements in the prognosis and early detection of BC, its morbidity and mortality remain very high and pose significant challenges, primarily due to the complex pathogenesis, multiple molecular subtypes, and high heterogeneity [2]. To effectively treat BC, it is imperative to discover new molecular biomarkers and develop novel diagnostic and therapeutic approaches.

Circular RNAs (circRNAs), a novel type of endogenous ncRNAs, unlike traditional linear RNAs, are a class of single-stranded RNA molecules with a covalently closed circular structure, which is mainly produced by reverse splicing of precursor mRNA (pre-mRNA) exons and lacks a 5⁣′-terminal cap and a 3⁣′-terminal polyadenylic acid tail (Poly A) [3], which makes them relatively stable in cells and not easily degraded by exonucleases [4]. As early as 1976, circRNA was first discovered by the Sanger team [5]. But at first, circRNAs did not attract much attention from the academic community [6]. Until the 21st century, as RNA-seq technology evolved dramatically, circRNAs were discovered in large numbers [7, 8], and the functionality of circRNAs began to be discovered and studied in depth [9]. The widespread expression of circRNA in many species has been demonstrated in numerous studies such as viral [10] and mammalian [11]. Meanwhile, there was a strong correlation found between circRNA and the emergence of certain malignancies, such as gastric cancer [12], BC [13], hepatocellular carcinoma [14], colorectal cancer [15], and other illnesses. Currently, the diversification of early diagnosis methods for BC and the pluralism of prognosis parameters provide a new evidence base and methods to screen for BC at an early stage and the selection of neoadjuvant chemotherapy regimens. The etiology and pathogenesis of BC are complex because tumors are highly heterogeneous and have various molecular subtypes. Therefore, making use of bioinformatics methods to find the molecular targets of BC and studying the detection methods and therapeutic drugs related to the targets has become a key link in the field of BC research.

circRNAs' primary function is to control gene expression by serving as the sponge for microRNAs [5]. For example, by adsorbing with miR-217-5p, CircEZH2 can upregulate KLF5 expression, thereby accentuating the carcinogenesis and metastasis of BC [16]. hsa_circ_0025202 has been shown to regulate tumor progression in BC cells by acting as a miRNA sponge for miR-182-5p [17].

We are primarily using the ceRNA mechanism of circRNA in this study. Using R software, we obtain the DEcircRNAs by using the circRNA database in BC tissue from the Gene Expression Omnibus (GEO). Use the database to predict miRNAs and mRNAs downstream of circRNA. The mRNAs were enriched according to Gene Ontology (GO) and Kyoto Encyclopedia of Genes and Genomes (KEGG) pathways, and the network of the ceRNAs was constructed. Verify the hsa_circ_0001314 expression and biological function by using in vitro experiments. The molecular mechanism by which hsa_circ_0001314 can act as a sponge of hsa-miR-548aj-3p, bind to hsa-miR-548aj-3p, activate the MAPK signaling pathway, and regulate the expression of MAPK8 and MAP3K1 genes. This study was aimed at identifying a novel therapeutic target for BC by focusing on circRNA-based ceRNA action, which not only facilitates a better understanding of the development mechanism of BC but also has important implications for the development of BC therapies.

## 2. Methods

### 2.1. Data Collection

Download the circRNA expression profile (GSE182471) and miRNA expression profile (GSE143564) in the GEO database (https://www.ncbi.nlm.nih.gov/) [18]. Based on the GPL21825 platform, the GSE182471 is a circRNA database that includes five BC tissue samples and five adjacent tissue samples. The GSE143564 is a miRNA database based on the GPL21572 platform that includes three BC tissue samples and three adjacent tissue samples.

### 2.2. Identification of circRNA

R is used to read the clinical and raw data for GSE182471. The probe and the gene symbol are matched based on the annotation file within the platform. The DEcircRNAs analysis was performed with the R, and the DEcircRNAs were identified using the *p* < 0.050 and |log_2_(fold change)| > 2. The volcano plot of the DEcircRNAs was displayed by the ggplot2 package in R. The heatmap plot of the DEcircRNAs was performed by heatmap plotting tools in Hiplot Pro (https://hiplot.com.cn/).

### 2.3. Prediction of miRNA and mRNA of circRNA

The target miRNAs of DEcircRNA were predicted using circBank (http://www.circbank.cn) [19]. For the differentially expressed miRNA (DEmiRNA) analysis, the R limma package was utilized. The DEmiRNAs in the GSE143564 dataset and these target miRNAs were intersected using a Venn diagram, and intersection miRNAs were subsequently obtained. To predict the interactions of miRNA and mRNA, miRDB (https://mirdb.org) [20] and TargetScan (https://www.targetscan.org/vert_80/) [21] were used. Then, we took the intersection of mRNAs between the miRDB and TargetScan, which we called DEmRNAs.

### 2.4. Functional Enrichment Analyses

The functional enrichment analysis of mRNAs was performed with the DAVID database (https://david.ncifcrf.gov) [22]. Three fields were included in the biological process (BP) section of the GO analysis: BPs, cellular component (CC), and molecular function (MF), while KEGG signaling pathways were used to further explore the pathway of DEmRNAs.

### 2.5. The ceRNA Network Construction

Using GeneMania (http://www.genemania.org) [23], the gene–gene interaction (GGI) network was created. The STRING database (https://cn.string-db.org) [24] was used to retrieve the interaction pairs between target genes, and the CytoNCA tool in Cytoscape was utilized to obtain the network's hub genes. The content of the analysis is betweenness centrality. Additionally, the PPI network was displayed using Cytoscape [25]. Based on the hub genes, the ceRNA network was built.

### 2.6. Expression Level and Survival Analysis of mRNAs

The online analytic system of the assistance for clinical bioinformatics, which is based on the TCGA database, examined the expression of hub genes in patients with BC. The UALCAN database analyzes the survival of hub genes in BC patients to explore whether the hub genes affect the survival rate of BC patients.

### 2.7. Tissue Sample Collection

From the Third Affiliated Hospital of Qiqihar Medical University, a total of 15 BC samples and 9 adjacent breast tissue samples were obtained. The study was approved by the Ethics Committee of Qiqihar Medical University, and informed consent was obtained from all patients.

### 2.8. Cell Culture and Transfection

Normal human mammary epithelial cells (MCF-10A) and human BC cells (MCF-7 and MDA-MB-231) were supplied by the Chinese Academy of Sciences Cell Bank (Shanghai, China). Fetal bovine serum (FBS, TBDscience, China) and penicillin–streptomycin solution (Biosharp, China) were added to RPMI 1640 (Procell, China) to cultivate the MCF-7 cells. MCF-10A and MDA-MB-231 were cultured using MCF-10A cell-specific medium (Procell, China) and DMEM high glucose medium (Procell, China), respectively. All cells were grown at 5% CO_2_ and 37°C. Si-hsa_circ_0001314-1, si-hsa_circ_0001314-2, and hsa-miR-548aj-3p for RNA interference in BC cells were designed and synthesized by GenePharma (Shanghai, China). Si-hsa_circ_0001314-1, si-hsa_circ_0001314-2, hsa-miR-548aj-3p, and negative control (NC) were transfected into MCF-7 cells. Then, the seeding of cells in six-well culture plates and transfecting with siRNA and NC using liposomal 6000 (Beyotime, China).  Table 1 contains a list of the siRNA and miRNA mimic synthesis sequences.

### 2.9. RT-qPCR

From tissue samples and cells, total RNA was extracted using TRIzol reagent (Tiangen, China), and reverse transcription of cDNA was performed using FastKing gDNA Dispelling RT SuperMix (Tiangen, China). RT-qPCR studies were conducted using Talent qPCR PreMix (Tiangen, China). Reference genes for GAPDH and U6 were used. The 2^−ΔΔCT^ method was employed to calculate the relative expression levels. Specific primer sequences refer to Table 2. To investigate the association between circRNA and miRNA (hsa_circ_0001314 and hsa-miR-548aj-3p), Pearson's correlation coefficients were employed.

### 2.10. Cell Counting Kit-8 (CCK-8) Proliferation Assay

The transfected BC cells will continue to be cultured under appropriate conditions for 24, 48, or 72 h. Then, add 10 *μ*L of CCK-8 reagent (Biosharp, China) to each well following the kit instructions. Using a microplate reader, the absorbance of the cells was determined at 450 nm after they had been incubated for 1 h at 37°C.

### 2.11. Wound Healing Assay

In six-well plates, transfected MCF-7 cells were cultivated until 80% confluence was attained. Using a 200 *μ*L sterile pipette tip, the cell surface was scraped. After PBS washes to remove cell debris, followed by culture at 37°C with fresh medium. Cells were visualized and photographed using a microscope (Olympus, Japan) at 0, 24, and 48 h. ImageJ was used to estimate scratch areas and calculate the relative migration rate.

### 2.12. Transwell Invasion Assay

Matrigel (ABW, China)-covered upper chamber was seeded with cells. The lower chamber was filled with a medium containing 10% FBS, and it was incubated for 48 h. Four percent paraformaldehyde is used to fix cells for 15 min, and 0.1% crystal violet is used to dye them for 10 min. Then, a cotton swab was used to wipe the top chamber. Cells were visualized and counted using a microscope.

### 2.13. Cell Apoptosis Assay

Apoptosis was assessed using the V-FITC/PI Apoptosis Assay Kit (Biosharp, China). Following trypsinization, the cells were resuspended in 250 *μ*L of binding buffer after being washed with cold PBS. Add 5 *μ*L of Annexin V-FITC and 10 *μ*L of PI, then incubate for 10 min at room temperature. Apoptosis was detected using a FACScalibur flow cytometer (BD Biosciences, United States).

### 2.14. Statistical Analysis

Triplicates of each experiment were run. ImageJ software and GraphPad Prism 9.5.1 software were used for statistical analysis of image production and experimental data. A *T*-test was used for univariate variable comparison, and variance analysis was used for data analysis between multiple groups. A possibility of *p* < 0.050 reflects a significant difference.

## 3. Results

### 3.1. Differentially Expressed circRNAs

**Figure 1:**
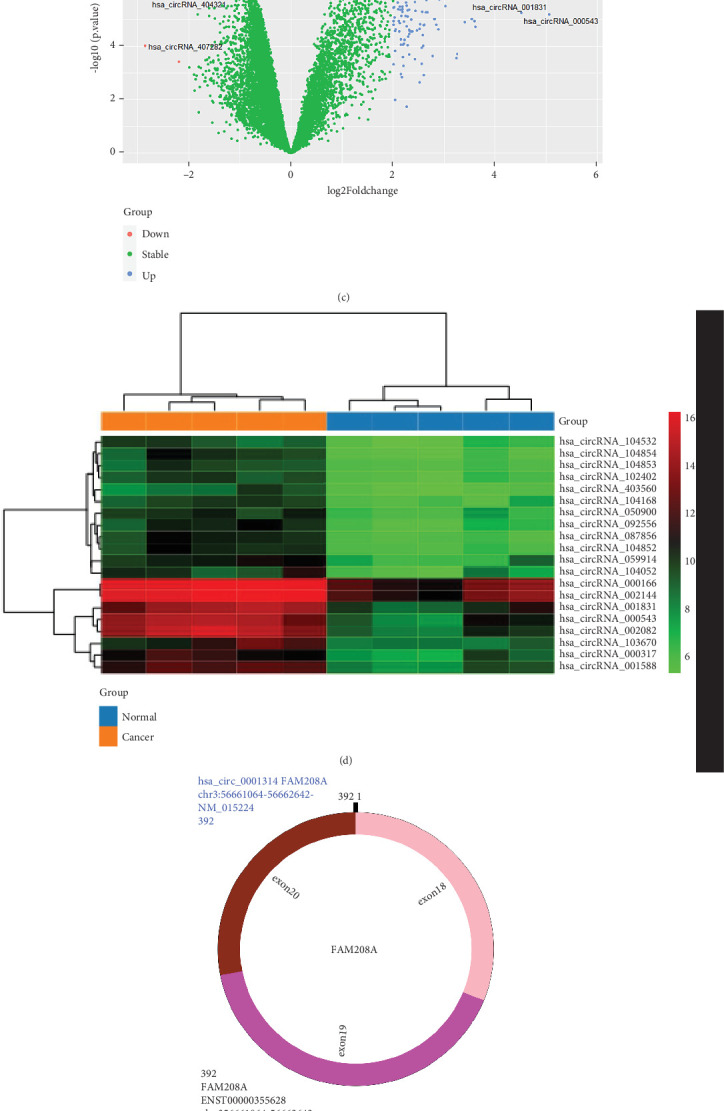
Identification of the DEcircRNAs, DEmiRNAs, and DEmRNAs. (a) Normalization of circRNA dataset. (b) Normalization of miRNA dataset. (c) Volcano plot of DEcircRNAs. (d) Heatmap of DEcircRNAs. (e) Structural patterns of hsa_circ_0001314. (f) Venn diagram of the intersection between target miRNAs and the DEmiRNA in the GSE143564 dataset. (g) Venn diagram of the intersection between target mRNAs from the miRDB and TargetScan predictions. (h) The binding site of hsa_circ_0001314 with hsa-miR-548aj-3p.

The datasets of GSE182471 and GSE143564 downloaded from the GEO database have been standardized (Figures 1(a) and 1(b)). Following the analysis, 142 DEcircRNAs were discovered, four of which had downregulation and 138 of which had upregulation. As demonstrated by the DEcircRNAs' heatmaps and volcano plot in Figures 1(c) and 1(d), one of the circRNAs that is most significantly upregulated in BC tissues is hsa_circ_001831. Finally, hsa_circ_001831 was regarded as the study's final DEcircRNA. The hsa_circ_001831 is converted into a universal name hsa_circ_0001314 based on circBase [26]. The structure pattern diagram of hsa_circ_0001314 (Figure 1(e)) was shown by CircPrimer 2.0 [27].

### 3.2. Prediction of miRNA and mRNA of circRNA

Through analysis and screening of the GEO database (GSE143564), a total of 4422 DEmiRNAs were obtained. Through predictions generated by the circBank database, 25 miRNAs were identified as binding to hsa_circ_0001314. Subsequently, as shown in Figure 1(f), a Venn diagram was used to intersect the 4422 DEmiRNAs with the 25 target miRNAs, yielding 23 intersecting miRNAs. Among them, hsa-miR-548aj-3p (*p* = 2.41e − 03) was significantly downregulated in BC tissues. CircMir displayed the binding site of hsa_circ_0001314 with hsa-miR-548aj-3p (Figure 1(h)). Therefore, hsa-miR-548aj-3p was selected as the downstream miRNA of hsa_circ_0001314 for subsequent research.

Using miRDB and TargetScan, the interactions between miRNA and mRNA were predicted. Then, as illustrated in Figure 1(g), the DEmRNAs of the miRDB and the TargetScan were intersected using the Venn diagram, yielding 360 intersection mRNAs.

### 3.3. GO and KEGG Pathway Enrichment Analyses

**Figure 2:**
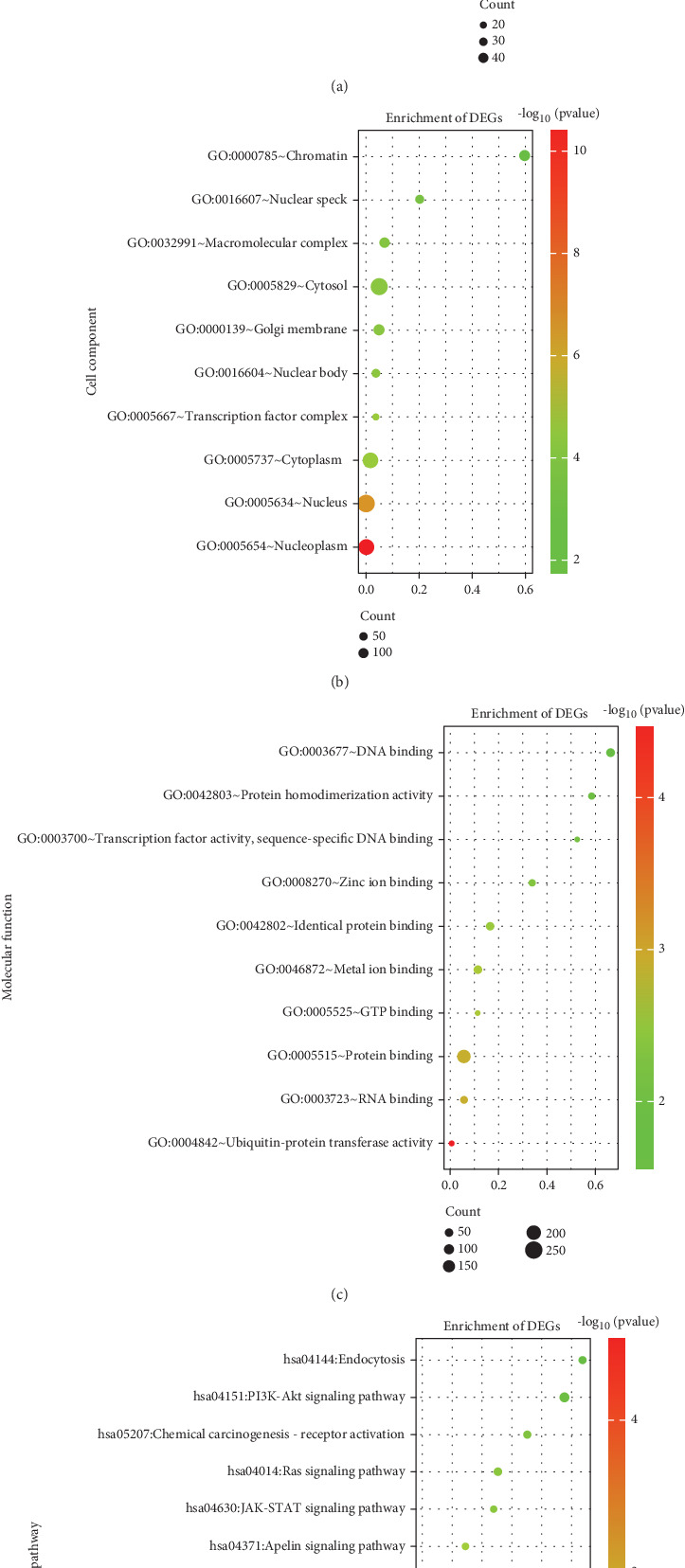
GO and KEGG pathway enrichment analyses. (a–c) The dot plot of Top 10 GO analysis. (d) The dot plot of Top 10 KEGG pathway analysis.

GO and KEGG analyses were performed on the 360 intersecting mRNAs to assess the role of the DEmRNAs in the network (Figure 2). The findings of the GO analysis demonstrated that the regulation of transcription from the RNA Polymerase II promoter and the negative regulation of transcription from the RNA Polymerase II promoter were the primary areas of enrichment for changes in BPs of DEmRNAs. The nucleus, cytoplasm, and nucleoplasm showed the greatest enrichment in changes in the CC of DEmRNAs. Protein binding and metal ion binding exhibited a high enrichment in the MF changes of DEmRNAs. The DEmRNAs were primarily abundant in the pathways related to cancer, the MAPK signaling system, the PI3K-Akt signaling pathway, the signaling pathways regulating pluripotency of stem cells, and the Ras signaling pathway, according to KEGG pathway analysis. Most of the pathways identified above are closely correlated with cancer progression. After analysis, MAPK signaling was the most significant number of enriched genes. The MAPK signaling pathway is one of the fundamental pathways of mammalian cells, as demonstrated by numerous research studies, and is closely related to important physiological activities such as cell proliferation, differentiation, apoptosis, and angiogenesis. Therefore, subsequent research will concentrate on the genes that are enriched in the MAPK signaling pathway. The KEGG analysis of partial results is listed in Table 3.

### 3.4. Construction of ceRNA Network

**Figure 3:**
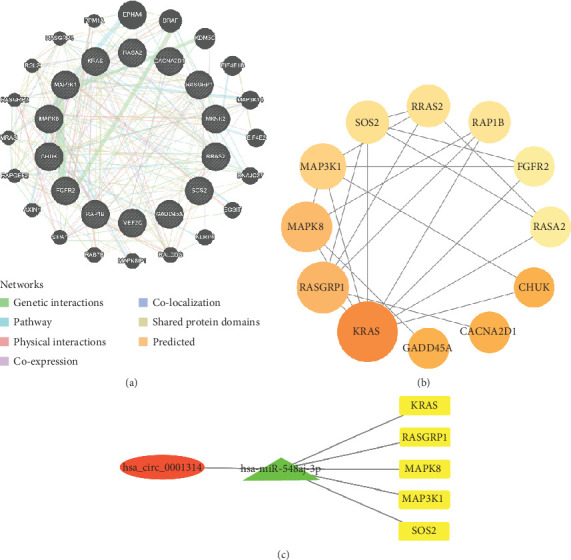
Construction of the network. (a) The GGI network of mRNAs. (b) The PPI network of mRNAs. (c) Cytoscape software was applied to create a visualization of the ceRNA network, which included one circRNA (hsa_circ_0001314), one miRNA (hsa-miR-548aj-3p), and five mRNAs (KRAS, RASGRP1, MAPK8, MAP3K1, and SOS2) (red: circRNA; green: miRNA; yellow: mRNAs).

The GeneMania database was used to construct the GGI network of the mRNAs (Figure 3(a)). These genes were closely related to genetic interactions, pathways, physical interactions, coexpression, colocalization, shared protein domains, etc. The STRING database and Cytoscape were used to construct the PPI network to perform a topological analysis of the network (Figure 3(b)). PPI network results showed that the Top 5 hub genes were KRAS, RASGRP1, MAPK8, MAP3K1, and SOS2 (Table 4).

The hub genes were chosen to form a circRNA-miRNA-mRNA regulatory network for subsequent investigation. Then, a ceRNA network was built (Figure 3(c)), which included one circRNA (hsa_circ_0001314), one miRNA (hsa-miR-548aj-3p), and five mRNAs (KRAS, RASGRP1, MAPK8, MAP3K1, and SOS2).

### 3.5. The Expression and Survival Analysis of mRNAs

**Figure 4:**
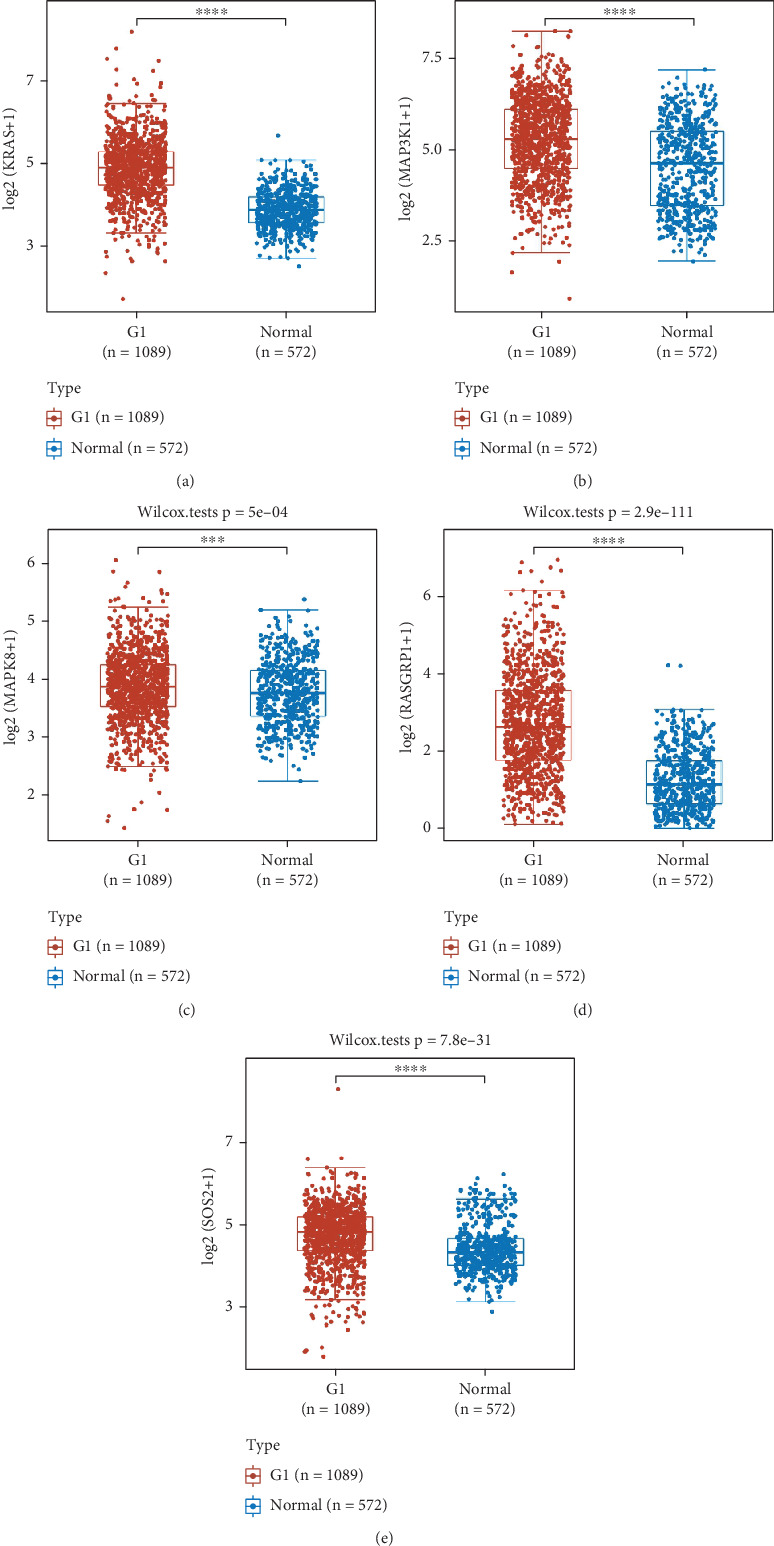
The expression of five hub genes in BC patients was analyzed (the G1 group was BC tissue, and the normal group was normal breast tissue; ⁣^∗∗∗^*p* < 0.001; ⁣^∗∗∗∗^*p* < 0.0001). (a) KRAS; (b) MAP3K1; (c) MAPK8; (d) RASGRP1; (e) SOS2.

The expression of five hub genes in BC patients was analyzed (Figure 4). The findings demonstrated that the gene expression of KRAS (*p* = 1.1e − 149), MAP3K1 (*p* = 5.7e − 29), MAPK8 (*p* = 5e − 04), RASGRP1 (*p* = 2.9e − 111), and SOS2 (*p* = 7.8e − 31) was considerably upregulated in BC as compared to the normal tissue.

**Figure 5:**
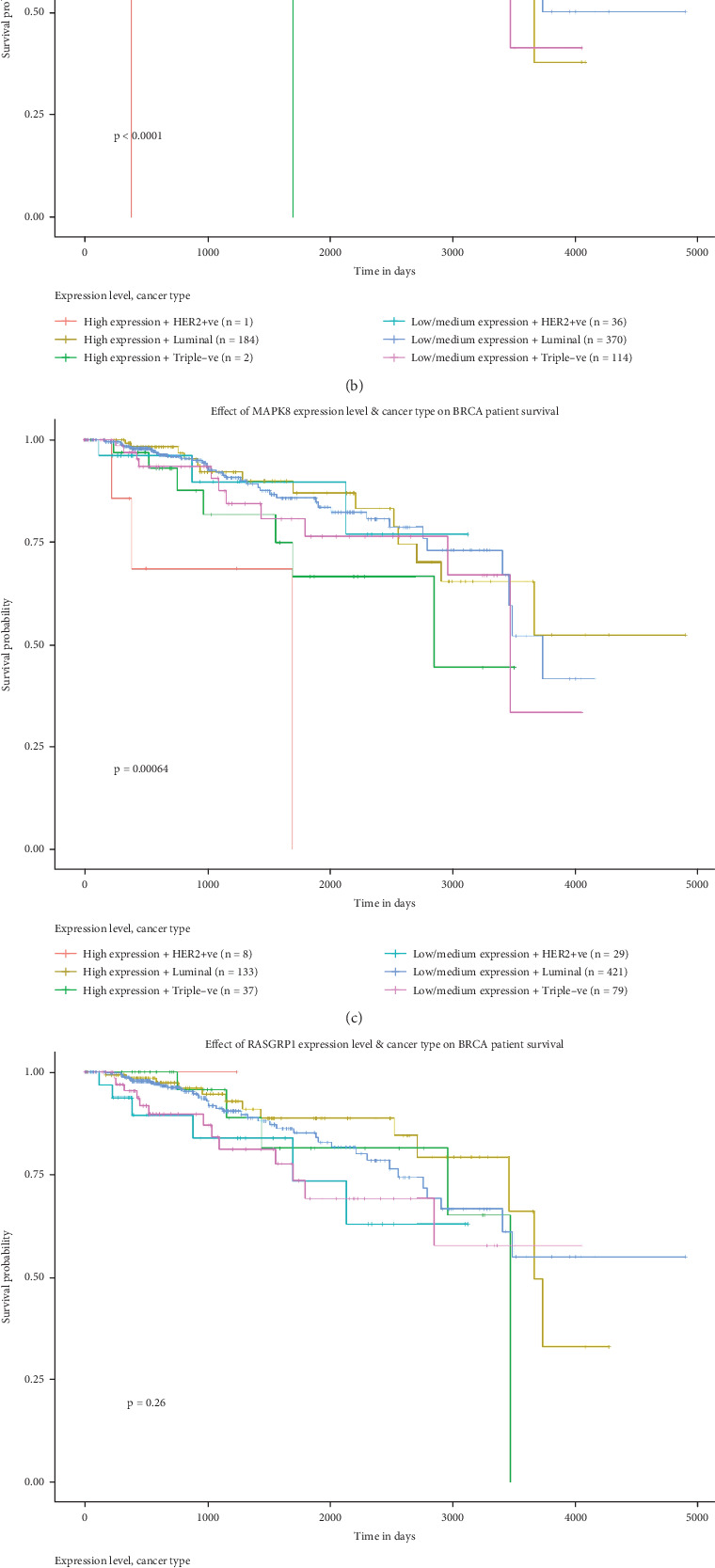
The survival rate of the five hub genes in BC patients was analyzed. (a) KRAS; (b) MAP3K1; (c) MAPK8; (d) RASGRP1; (e) SOS2.

To further explore whether the hub genes influence the prognostic survival in BC patients, the survival rate of the five hub genes in BC patients was analyzed using UALCAN. By survival analysis, the expression of KRAS, MAP3K1, and MAPK8 genes correlated with survival rate, while BC patients with low expression of KRAS (*p* < 0.0001), MAP3K1 (*p* < 0.0001), and MAPK8 (*p* = 0.00064) had a significantly higher survival rate than those with high expression, while the expression of RASGRP1 gene (*p* = 0.260) and SOS2 gene (*p* = 0.210) showed no significant relationship with survival rate (Figure 5).

### 3.6. hsa_circ_0001314 Is Upregulated in BC

**Figure 6:**
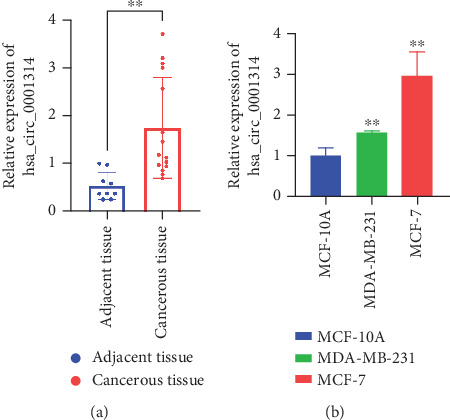
The level of hsa_circ_0001314 in BC tissue and cell is significantly increased. (a) RT-qPCR revealed that the level of hsa_circ_0001314 in BC tissues was higher than that in adjacent tissues (⁣^∗∗^*p* < 0.010). (b) Compared with MCF-10A, the expression of hsa_circ_0001314 in BC cells (MCF-7 and MDA-MB-231) was increased significantly (⁣^∗∗^*p* < 0.010).

To confirm the differential expression of hsa_circ_0001314 in BC, the expression of hsa_circ_0001314 in BC tissue and normal breast tissue, human BC cells (MCF-7 and MDA-MB-231), and normal human breast cells (MCF-10A) was detected by RT-qPCR (Figure 6). The results were consistent with bioinformatics predictions. hsa_circ_0001314 was highly expressed in BC tissues (*p* = 0.0028, *p* < 0.010) and BC cells (*p* = 0.0069, *p* < 0.010; *p* = 0.0053, *p* < 0.010), especially in MCF-7 cells, so MCF-7 cells were chosen for further investigations.

### 3.7. hsa_circ_0001314 Knockdown Suppressed BC Cell Proliferation, Migration, Invasion, and Promoted Apoptosis

**Figure 7:**
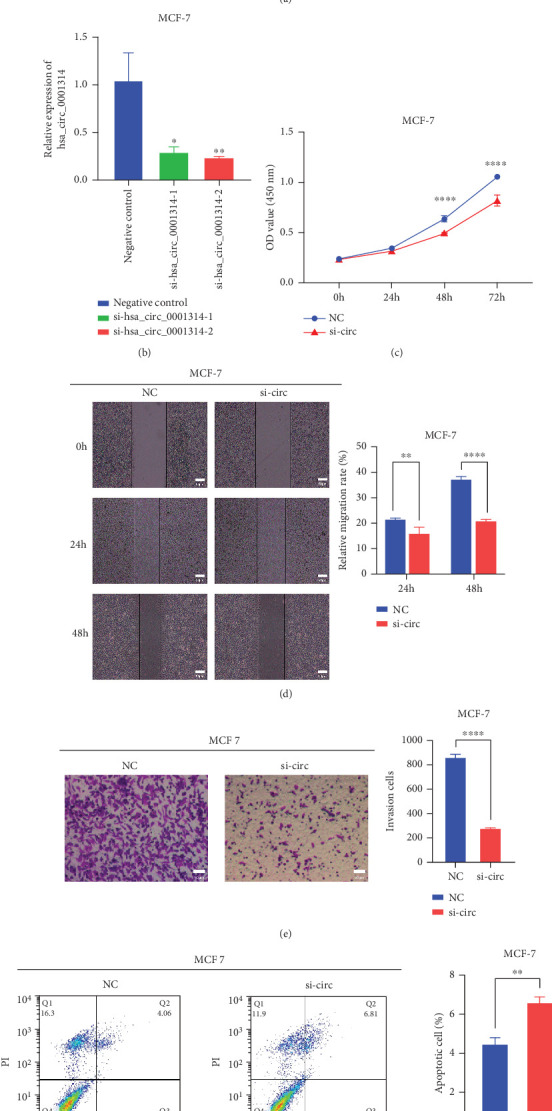
Effect of hsa_circ_0001314 on the proliferative and metastatic capacity of BC cells. (a) Fluorescence signals of the transfected cells were observed under a fluorescence microscope. (b) The siRNAs of hsa_circ_0001314 were transfected to BC cells. Compared with si-hsa_circ_0001314-1, si-hsa_circ_0001314-2 had higher interference efficiency (⁣^∗^*p* < 0.050, ⁣^∗∗^*p* < 0.010). (c) CCK-8 assay revealed that the knockdown of hsa_circ_001314 significantly reduced the proliferation of BC cells (⁣^∗∗∗∗^*p* < 0.0001). (d) Wound healing assay revealed that the knockdown of hsa_circ_001314 dramatically inhibited the migration of BC cells (⁣^∗∗^*p* < 0.010, ⁣^∗∗∗∗^*p* < 0.0001). (e) Transwell assay revealed that the knockdown of hsa_circ_001314 dramatically inhibited the invasion of BC cells (⁣^∗∗∗∗^*p* < 0.0001). (f) Flow cytometry assay revealed that the knockdown of hsa_circ_001314 significantly induced cell apoptosis in BC cells (⁣^∗∗^*p* < 0.010).

Reduce the expression of hsa_circ_0001314 in MCF-7 to investigate the impact of hsa_circ_0001314 on the biological function of BC cells. After 12 h of transfection, the distribution of intracellular fluorescence signals in MCF-7 was examined using fluorescence microscopy (Figure 7(a)). RT-qPCR was used to detect the expression of hsa_circ_0001314 and verify the transfection efficiency. The outcomes demonstrated that, in comparison to the NC, the expression levels of hsa_circ_0001314 in MCF-7 transfected with si-hsa_circ_0001314-1 and si-hsa_circ_0001314-2 were lower. In addition, the expression level of hsa_circ_0001314 in MCF-7 transfected with si-hsa_circ_0001314-2 was lower than that of transfected with si-hsa_circ_0001314-1; that is, si-hsa_circ_0001314-2 had higher interference efficiency. The si-hsa_circ_001314-2 with better transfection efficiency was chosen for further investigation (Figure 7(b)).

The CCK-8 assay revealed that the knockdown of hsa_circ_001314 significantly reduced the proliferation of BC cells (Figure 7(c)). Wound healing and transwell assays revealed that knocking down hsa_circ_001314 dramatically inhibited the invasion and migration ability of BC cells (Figures 7(d) and 7(e)). Flow cytometry assay revealed that the knockdown of hsa_circ_001314 significantly induced cell apoptosis in BC cells (Figure 7(f)). These results demonstrated that knocking down hsa_circ_001314 inhibited BC cell proliferation, migration, and invasion while increasing apoptosis.

### 3.8. hsa_circ_0001314 Binds to hsa-miR-548aj-3p in BC and Regulate the Expression of MAPK8/MAP3K1

**Figure 8:**
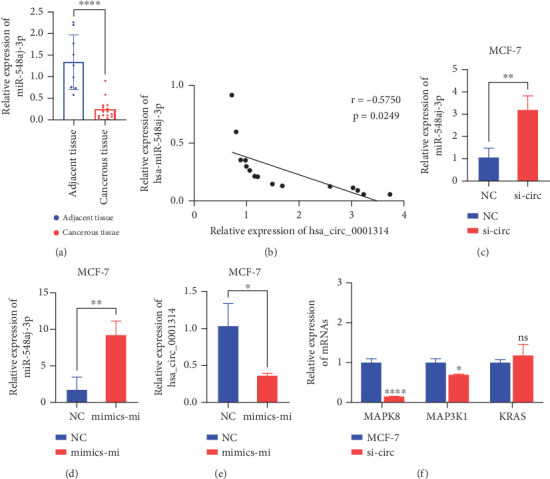
hsa_circ_0001314 binds to hsa-miR-548aj-3p in BC and regulates the expression of MAPK8/MAP3K1. (a) RT-qPCR revealed that the level of hsa-miR-548aj-3p in BC tissues was lower than that in adjacent tissues (⁣^∗∗∗∗^*p* < 0.0001). (b) Correlation analysis indicated that the expression levels of hsa_circ_0001314 and hsa-miR-548aj-3p were negatively correlated in BC tissues (*r* = −0.5750, *p* = 0.0249). (c) After the knockdown of hsa_circ_0001314, the expression level of hsa-miR-548aj-3p was higher than that in the NC (⁣^∗∗^*p* < 0.010). (d) Transfection efficiency was verified after transfection of hsa-miR-548aj-3p mimics in MCF-7 (⁣^∗∗^*p* < 0.010). (e) After overexpression of hsa-miR-548aj-3p, the expression level of hsa_circ_0001314 was lower than that of NC (⁣^∗^*p* < 0.050). (f) Compared with the expression in MCF-7, the expression of MAPK8 and MAP3K1 genes was significantly reduced after knockdown hsa_circ_0001314, and there was no significant change in KRAS (⁣^∗^*p* < 0.050, ⁣^∗∗∗∗^*p* < 0.0001; the ns indicates that the difference is not significant).

hsa-miR-548aj-3p expression in BC tissues was assessed by RT-qPCR. The findings illustrated that hsa-miR-548aj-3p was significantly downregulated (Figure 8(a)). Furthermore, hsa-miR-548aj-3p expression was found to be negatively linked with hsa_circ_0001314 expression in BC tissues (Figure 8(b)). These investigations preliminarily demonstrated that hsa_circ_0001314 could be bound to hsa-miR-548aj-3p in BC.

To further confirm that hsa-miR-548aj-3p is the miRNA bound by hsa_circ_0001314, si-hsa_circ_0001314-2 was transfected into MCF-7 cells to knock down the expression level of hsa_circ_0001314, and the expression of hsa-miR-548aj-3p was detected by RT-qPCR. The results showed that, after the knockdown of hsa_circ_0001314, the expression level of hsa-miR-548aj-3p was higher than that in the NC group (Figure 8(c)). Furthermore, hsa-miR-548aj-3p mimics were transfected into MCF-7 cells to overexpress hsa-miR-548aj-3p, with this group designated as the mimics-mi group. The transfection efficiency was verified using RT-qPCR. The results indicated that the expression level of hsa-miR-548aj-3p in the mimics-mi group was higher than that in the NC group, confirming successful transfection and suitability for subsequent experiments (Figure 8(d)). RT-qPCR was employed to detect the expression of hsa_circ_0001314. The results revealed that, compared to the NC group, overexpression of hsa-miR-548aj-3p led to a decrease in the expression level of hsa_circ_0001314 (Figure 8(e)). These findings demonstrate that the knockdown of hsa_circ_0001314 increases the expression of hsa-miR-548aj-3p, while overexpression of hsa-miR-548aj-3p decreases the expression of hsa_circ_0001314. In conclusion, hsa_circ_0001314 functions as a sponge for hsa-miR-548aj-3p, targeting and binding to it.

According to the prediction of the database, hsa-miR-548aj-3p can target KRAS, RASGRP1, MAPK8, MAP3K1, and SOS2 genes. Because the expression of MAPK8, MAP3K1, and KRAS genes is significantly upregulated in BC and is associated with the survival of BC patients, MAPK8, MAP3K1, and KRAS genes were selected for follow-up research. MCF-7 was transfected with si-hsa_circ_0001314-2 for knockdown of hsa_circ_0001314 expression, and the expression of MAPK8, MAP3K1, and KRAS genes was detected by RT-qPCR (Figure 8(f)). The results showed that compared with the expression in MCF-7, the expression of MAPK8 and MAP3K1 genes was significantly reduced after knockdown hsa_circ_0001314, and there was no significant change in KRAS. These suggested that hsa_circ_0001314 could regulate the expression of MAPK8 and MAP3K1.

## 4. Discussion

As one of the noncoding RNAs, the circRNA is mainly formed by the pre-mRNA through “reverse splicing,” which has a variety of functions and special properties [28]. circRNA is mostly found in the cytoplasm, and very little of it is found in the nucleus [29]. Compared to its linear counterparts, circRNA has been shown in certain studies to have longer half-lives [30]. Due to their high expression levels, structural stability, and diverse biological functions, circRNAs are increasingly attracting the attention of researchers. With the rapid advancements in sequencing and bioinformatics technologies, preliminary studies have revealed that circRNAs play crucial roles in carcinogenesis, metastasis, or apoptosis in BC, and potential new therapeutic targets for the treatment of this disease may be offered by them. However, the specific functions of most circRNAs and their molecular mechanisms in BC remain largely unexplored. Therefore, identifying novel circRNAs with biological functions would contribute to elucidating the mechanisms underlying the development of BC, providing a solid theoretical basis for the diagnosis and treatment of this disease.

The circRNA dataset (GSE182471) was used in this study to successfully screen for the hsa_circ_0001314, whose expression was upregulated in BC. hsa_circ_0001314 was validated to be highly expressed in BC tissues and cells by RT-qPCR, which is consistent with the predicted results from bioinformatics. Therefore, it is speculated that hsa_circ_0001314 may be involved in the onset and progression of BC, and its cell function and related mechanisms will be analyzed and verified. According to research, hsa_circ_0001314 expression knockdown can dramatically reduce BC cell proliferation, migration, and invasion while increasing apoptosis.

In addition, some recent studies have suggested that circRNA comprises characters with several binding sites of miRNA. Through the ceRNA mechanism, which binds to particular miRNAs to control gene expression, it can function as a sponge, such as circBACH2 (hsa_circ_0001625), which functions as an hsa-miR-944 sponge to enhance HNRNPC expression and stimulate BC cell proliferation [31]. The circ_0008039 regulates SKA3 by sponging miR-140-3p [32]. CircCD44 stimulates the growth of TNBC by focusing on the miR-502-5p-KRAS axis and C-myc [33]. Therefore, it is considered that hsa_circ_0001314 may also promote the malignant progression of BC through this mechanism. Subsequently, the regulatory mechanism of hsa_circ_0001314 in BC is investigated. Initially, bioinformatics analysis predicted that hsa_circ_0001314 targets and binds to hsa-miR-548aj-3p. It was confirmed by RT-qPCR that hsa-miR-548aj-3p is lowly expressed in BC tissues, which contrasts with the high expression of hsa_circ_0001314 in the BC tissues. Pearson's analysis revealed a significant negative correlation between the expression levels of hsa_circ_0001314 and hsa-miR-548aj-3p in BC tissues. Therefore, it can be tentatively concluded that hsa_circ_0001314 targets and binds to hsa-miR-548aj-3p. hsa_circ_0001314 was knocked down and hsa-miR-548aj-3p was overexpressed in MCF-7 cells, respectively, and the expression levels of hsa-miR-548aj-3p and hsa_circ_0001314 were detected by RT-qPCR. The results showed that knocking down hsa_circ_0001314 increased the expression of hsa-miR-548aj-3p while overexpressing hsa-miR-548aj-3p decreased the expression of hsa_circ_0001314. In summary, it is established that hsa_circ_0001314 functions as a sponge for hsa-miR-548aj-3p, enabling targeted binding to it.

Next, bioinformatics methods were employed to predict the potential target mRNAs of hsa-miR-548aj-3p, and functional enrichment analysis was further conducted on these predicted mRNAs. Analysis indicated that the majority of genes were enriched in the MAPK signaling pathway, which is one of the core pathways in mammalian cells and is closely associated with crucial physiological processes such as cell growth, differentiation, apoptosis, and angiogenesis. Therefore, genes enriched in the MAPK signaling pathway were selected as the main focus of subsequent research. Fifteen DEmRNAs enriched in the MAPK signaling pathway were selected for constructing both the GGI network and the PPI network. KRAS, RASGRP1, MAPK8, MAP3K1, and SOS2, which ranked in the Top 5 based on betweenness centrality, were identified as hub genes. These hub genes were then chosen to construct the ceRNA regulatory network involving hsa_circ_0001314-hsa-miR-548aj-3p-mRNAs. Bioinformatics analysis revealed that the KRAS, MAP3K1, and MAPK8 genes were significantly upregulated in BC and correlated with the prognosis and survival of BC patients, prompting their selection for further study. The expression of hsa_circ_0001314 was knocked down in MCF-7 cells, and the expression levels of KRAS, MAP3K1, and MAPK8 genes were detected by RT-qPCR. The results showed that, compared to their expression in MCF-7 cells, the expression of MAPK8 and MAP3K1 genes was significantly reduced after knocking down hsa_circ_0001314, while there was no significant change in KRAS gene expression. This suggests that hsa_circ_0001314 can regulate the expression of MAPK8 and MAP3K1 genes.

For the first time, hsa_circ_0001314 was discovered to be considerably overexpressed in BC tissues and cell lines in our investigation. Then, in vitro experiments confirmed that knocking down the expression level of hsa_circ_0001314 could significantly inhibit the proliferation, migration, and invasion of BC cells and promote the biological effect of apoptosis of BC cells. The molecular mechanism that hsa_circ_0001314 can act as a sponge of hsa-miR-548aj-3p and combine with hsa-miR-548aj-3p to activate MAPK signaling pathway, regulate the expression of MAPK8 and MAP3K1 genes, and exert biological effects. However, our study only preliminarily validated the interaction between hsa_circ_0001314 and hsa-miR-548aj-3p, which has some limitations. In the future, we will verify the interaction between hsa_circ_0001314 and hsa-miR-548aj-3p through a luciferase reporter assay to support the mechanism of ceRNA. Our main goal in the next phase of research is to verify the biological function of hsa_circ_0001314 through in vivo studies and further explore the role of hsa_circ_0001314 as a therapeutic target, hoping to improve the molecular mechanism of hsa_circ_0001314 in the occurrence and progression of BC.

## 5. Conclusions

In conclusion, hsa_circ_0001314 is highly expressed in BC tissues and cells. Significant inhibition of cell proliferation, migration, and invasion, as well as promotion of apoptosis, was achieved through knockdown of the hsa_circ_0001314 expression level, promoting apoptosis and constructing the ceRNA network for the hsa_circ_0001314-hsa-miR-548aj-3p-mRNAs. The molecular mechanism by which hsa_circ_0001314 can act as a sponge of hsa-miR-548aj-3p, bind to hsa-miR-548aj-3p, activate the MAPK signaling pathway, regulate the expression of MAPK8 and MAP3K1 genes, and play a biological role was preliminarily demonstrated. The research offers a new possible target for the identification and treatment of BC by elucidating the mechanism of action of hsa_circ_0001314 in BC for the first time.

## Figures and Tables

**Table 1:** Sequences of the synthesis.

**Target name**		**Sequences (5**⁣′**-3**⁣′**)**
Negative control	Sense	UUCUCCGAACGUGUCACGUTT
	Antisense	ACGUGACACGUUCGGAGAATT
si-hsa_circ_0001314-1	Sense	GGAACUAGGCAGGAAUAUCTT
	Antisense	GAUAUUCCUGCCUAGUUCCTT
si-hsa_circ_0001314-2	Sense	CUAGGCAGGAAUAUCUUAUTT
	Antisense	AUAAGAUAUUCCUGCCUAGTT
hsa-miR-548aj-3p	Sense	UAAAAACUGCAAUUACUUUUA
	Antisense	AAAGUAAUUGCAGUUUUUAUU

**Table 2:** Sequences of primers for RT-qPCR.

**Primer name**	**Primer sequences (5**⁣′**-3**⁣′**)**
hsa_circ_0001314	F: GAAACTAAGGAACTAGGCAGG
	R: CAAGCCAGGTATCTTGTGAATG
GAPDH	F: AGAAGGCTGGGGCTCATTTG
	R: GCAGGAGGCATTGCTGATGAT
hsa-miR-548aj-3p	F: CGCGCGTAAAAACTGCAATTA
	R: AGTGCAGGGTCCGAGGTATT
U6	F: CTCGCTTCGGCAGCACA
	R: AACGCTTCACGAATTTGCGT
KRAS	F: TAGGCAAGAGTGCCTTGACG
	R: CCCTCCCCAGTCCTCATGTA
MAP3K1	F: AGGTCGCACAGTGAAATCAG
	R: GTTTCCTCAGGGCTATATGGTG
MAPK8	F: GACGCCTTATGTAGTGACTCGC
	R: TCCTGGAAAGAGGATTTTGTGGC

**Table 3:** KEGG analysis of partial results.

**Terms**	**Count**	**p** ** value**	**Genes**
hsa05200: Pathways in cancer	21	4.52e − 03	SMAD2, GSK3B, SMAD4, HDAC2, CHUK, GADD45A, IL15, DLL1, RASGRP1, EPOR, GNAI1, AGT, MAPK8, CCND1, GNG7, COL4A5, KRAS, VHL, JAK2, SOS2, and FGFR2
hsa04010: MAPK signaling pathway	15	2.25e − 03	MEF2C, MAP3K1, CHUK, GADD45A, CACNA2D1, RRAS2, RASGRP1, RAP1B, MAPK8, RASA2, MKNK2, KRAS, LAMTOR3, SOS2, and FGFR2
hsa04151: PI3K-Akt signaling pathway	13	5.15e − 02	GSK3B, CHUK, YWHAZ, EPOR, CCND1, PPP2R5E, GNG7, COL4A5, KRAS, JAK2, SOS2, FGFR2, and MCL1
hsa04014: Ras signaling pathway	11	1.79e − 02	RAP1B, RAB5B, MAPK8, CHUK, GNG7, RASA2, RRAS2, KRAS, RASGRP1, SOS2, and FGFR2
hsa05224: Breast cancer	7	7.44e − 02	GSK3B, CCND1, GADD45A, TNFSF11, KRAS, DLL1, and SOS2

**Table 4:** The table shows the genes ranked by BC.

	**Nodes**	**Betweenness**
1	KRAS	41.0
2	RASGRP1	23.5
3	MAPK8	21.0
4	MAP3K1	12.5
5	SOS2	5.8333335
6	RRAS2	5.0
7	RAP1B	4.0
8	FGFR2	0.6666667
9	RASA2	0.5
10	GADD45A	0.0
11	CHUK	0.0
12	CACNA2D1	0.0

## Data Availability

Our study used a public online database. The data can be accessed by the following websites: https://www.cancer.gov/about-nci/organization/ccg/research/structural-genomics/tcga, https://www.ncbi.nlm.nih.gov/geo/query/acc.cgi?acc=GSE182471, and https://www.ncbi.nlm.nih.gov/geo/query/acc.cgi?acc=GSE143564.

## Nomenclature

BC: breast cancer
BPs: biological processes
CC: cellular component
ceRNA: competitive endogenous RNA
circRNA: circular RNA
DAVID: Database for Annotation, Visualization, and Integrated Discovery
DEcircRNAs: differentially expressed circRNAs
DEmiRNAs: differentially expressed miRNAs
GAPDH: glyceraldehyde-3-phosphate dehydrogenase
GEO: Gene Expression Omnibus
GGI: gene–gene interaction
GO: Gene Ontology
KEGG: Kyoto Encyclopedia of Genes and Genomes
MF: molecular function
miRNA: microRNA
mRNA: messenger RNA
PPI: protein–protein interaction
RT-qPCR: real-time quantitative PCR
STRING: Search Tool for the Retrieval of Interacting Genes
UALCAN: University of Alabama at Birmingham Cancer data analysis portal
